# Supplementary material for: Interfacial magnetic spin Hall effect in van der Waals Fe3GeTe2/MoTe2 heterostructure
Source: Nat Commun. 2024 Feb 7;15:1129. doi: 10.1038/s41467-024-45318-8 (PMC10847462; doi:10.1038/s41467-024-45318-8)
Supplement: Supplementary file 1 — Supplementary information [file 41467_2024_45318_MOESM1_ESM.pdf]

# Supplementary Information

# Interfacial magnetic spin Hall effect in van der Waals $Fe_3GeTe_2/MoTe_2$ heterostructure

Yudi Dai[1,9], Junlin Xiong[1,9], Yanfeng Ge[2,9], Bin Cheng[3]*, Lizheng Wang[1], Pengfei Wang[1], Zenglin Liu[1], Shengnan Yan[1], Cuiwei Zhang[4], Xianghan Xu[5], Youguo Shi[4], Sang-Wook Cheong[5], Cong Xiao[6,7,8]*, Shengyuan A. Yang[6], Shi-Jun Liang[1]*, Feng Miao[1]*

[1]National Laboratory of Solid State Microstructures, Institute of Brain-Inspired Intelligence, School of Physics, Collaborative Innovation Center of Advanced Microstructures, Nanjing University, Nanjing 210093, China.

[2]Research Laboratory for Quantum Materials, Singapore University of Technology and Design, Singapore, Singapore.

[3]Institute of Interdisciplinary Physical Sciences, School of Science, Nanjing University of Science and Technology, Nanjing 210094, China.

[4]Institute of Physics, Chinese Academy of Sciences, Beijing 100190, China.

[5]Center for Quantum Materials Synthesis and Department of Physics and Astronomy, Rutgers, The State University of New Jersey, Piscataway, NJ, 08854, USA.

[6]Institute of Applied Physics and Materials Engineering, University of Macau, Taipa, Macau SAR, China.

[7]Department of Physics, University of Hong Kong, Hong Kong, China.

[8]HKU-UCAS Joint Institute of Theoretical and Computational Physics at Hong Kong, Hong Kong, China.

[9]Contribute equally to this work.

*Correspondence Email:

bincheng@njust.edu.cn; congxiao@um.edu.mo; sjliang@nju.edu.cn; miao@nju.edu.cn

**Supplementary Note 1. Spin diffusion length of $MoTe_2$**

To obtain the spin diffusion length ($L_{sf}$) of $MoTe_2$, we performed length-dependence transport experiments in a $MoTe_2$ H-bar structure (inset of Supplementary Fig. 1). In this setup, spin current generated via spin Hall effect at the local Hall cross diffuses along the channel and generates charge imbalance induced by inverse spin Hall effect at the nonlocal Hall cross. The ratio between channel length and channel width is larger than three, ensuring negligible ohmic contribution to the non-local signal. Supplementary Figure 1 shows the length-dependent $R_{SH}$ and a fit to $R_{SH} \propto e^{-L/L_{sf}}$ (ref.[1]), which yields $L_{sf} = 1.6\ \mu m$.

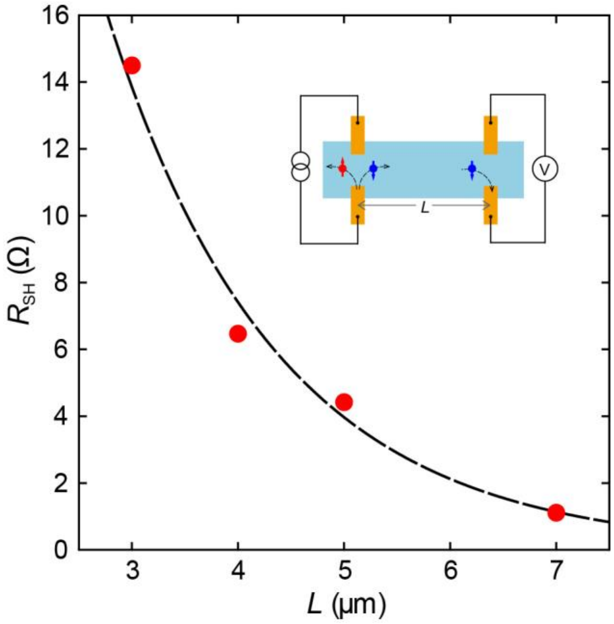


**Supplementary Fig. 1** Length-dependence $R_{SH}$ at 1.5 K measured by configuration shown in the inset. $L$ is the center-to-center distance between current injectors and voltage probes. The polarization direction of spin illustrated in inset is perpendicular to the transport plane.

**Supplementary Note 2. Exclusion of contribution from Rashba-Edelstein effect**

We perform a careful analysis to exclude the contribution of Rashba-Edelstein effect to the charge-to-spin conversion we observed. The specific analysis is summarized below:

(1) Rashba-Edelstein effect can only induce in-plane nonequilibrium spin density as a consequence of an applied electric field, in which the direction of spin polarization is in-plane and perpendicular to the direction of electric field. According to such configuration, when we apply a charge current along x-axis in heterostructure, the spins generated from Rashba effect is polarized along y-axis (i.e., $S_y$) and also diffuse along y-axis, denoted as $j_y^{S_y}$. $MoTe_2$-based non-local spin detector (with the detection Hall bar along x-axis) used in our FGT/$MoTe_2$ device cannot detect the resulting spin current, instead can only detect the spin current along y-axis with spin polarization along z-axis (corresponding to $\sigma_{yx}^{z}$) via inverse spin Hall effect. This is because $T_d$-$MoTe_2$ only allows six nonzero spin Hall conductivity tensor elements with orthogonal charge current, spin current and spin polarization[2]. This result indicates that the

Rashaba-Edelstein effect cannot contribute to the charge-to-spin conversion observed in our work.

(2) The spin current generated over FGT/$MoTe_2$ heterostructure region in our device is $\mathcal{T}$-odd, and thus the direction of generated spin current is locked to the magnetization of FGT. However, the Rashba effect is only determined by the SOC and inversion symmetry breaking at the interface, and is not relevant to the direction of magnetization. Thus, the $\mathcal{T}$-odd spin current in our work cannot be induced by Rashba effect.

**Supplementary Note 3. Symmetry analysis of FGT and FGT/MoTe₂ heterostructure**

The bulk structure of $Fe_3GeTe_2$ (FGT) belongs to the Laue group of 6/mm'm' and the two-dimensional structure of FGT belongs to the Laue group of -31m', both of which only allow $\sigma^z_{xx}$, $\sigma^z_{yy}$, and $\sigma^z_{zz}$ in $\mathcal{T}$-odd part of the spin-conductivity tensor $\sigma^z$ to have non-zero values, as shown in the following Supplementary Table 1. Meanwhile, the space group of bulk $T_d$-$MoTe_2$ is $Pmn2_1$ (no. 31), which has two mirror symmetries, i.e., a pure mirror symmetry and a glide mirror symmetry. When those two materials are stacked together, the glide mirror is not preserved at the interface of FGT/$MoTe_2$ heterostructure. The intentional or non-intentional alignment between the mirror planes of both FGT and $MoTe_2$ will determine the existence of $\mathcal{T}$-odd spin Hall conductivity $\sigma^z_{yx}$. When the mirror planes of both FGT and $MoTe_2$ are not intentionally aligned, the heterointerface has no mirror symmetry and the resulting symmetry belongs to the Laue group of -1. In this way, all relevant symmetries are broken and nonzero $\mathcal{T}$-odd spin Hall conductivity $\sigma^z_{yx}$ is allowed, as shown in our work. While the mirror planes of both FGT and $MoTe_2$ are intentionally aligned, the mirror plane perpendicular to the film plane is preserved and the heterostructure symmetry belongs to 2'/m', which forbids the $\mathcal{T}$-odd spin Hall conductivity $\sigma^z_{yx}$. All symmetry analysis we carried out is based on the Neumann's principle[3-5].

| Laue group | $\sigma^x$ | $\sigma^y$ | $\sigma^z$ |
|---|---|---|---|
| 6/mm'm' | $\begin{pmatrix} 0 & 0 & \sigma^x_{xz} \\ 0 & 0 & 0 \\ \sigma^x_{zx} & 0 & 0 \end{pmatrix}$ | $\begin{pmatrix} 0 & 0 & 0 \\ 0 & 0 & \sigma^y_{yz} \\ 0 & \sigma^y_{zy} & 0 \end{pmatrix}$ | $\begin{pmatrix} \sigma^z_{xx} & 0 & 0 \\ 0 & \sigma^z_{yy} & 0 \\ 0 & 0 & \sigma^z_{zz} \end{pmatrix}$ |
| -31m' | $\begin{pmatrix} \sigma^x_{xx} & 0 & \sigma^x_{xz} \\ 0 & \sigma^x_{yy} & 0 \\ \sigma^x_{zx} & 0 & 0 \end{pmatrix}$ | $\begin{pmatrix} 0 & \sigma^y_{xy} & 0 \\ \sigma^y_{yx} & 0 & \sigma^y_{yz} \\ 0 & \sigma^y_{zy} & 0 \end{pmatrix}$ | $\begin{pmatrix} \sigma^z_{xx} & 0 & 0 \\ 0 & \sigma^z_{yy} & 0 \\ 0 & 0 & \sigma^z_{zz} \end{pmatrix}$ |

**Supplementary Table 1.** Symmetry-allowed time-odd spin Hall conductivity tensors in FGT. The spin Hall conductivity tensor $\sigma^{\gamma}_{\alpha\beta}$ is a third-order tensor, where $\beta$ and $\alpha$ are the directions of charge current and spin current, respectively. $\gamma$ is spin polarization.

**Supplementary Note 4. Reproducibility of the interfacial-MSHE**

We fabricated five different devices and all the devices have the similar behaviors of magnetic spin Hall effect, as shown in Supplementary Fig. 2.

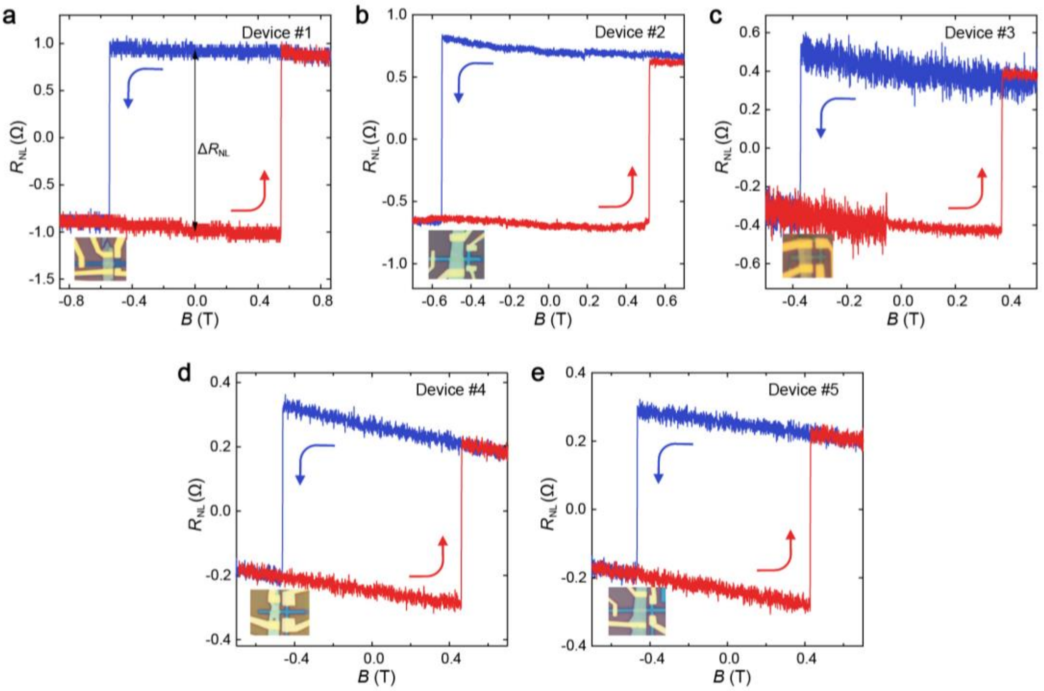


**Supplementary Fig. 2** Nonlocal transport measurements of the interfacial-MSHE in five different devices (Device #1-Device #5). The resistance jumps in the $R_{\mathrm{NL}}$–$B$ hysteresis loop are denoted as $\Delta R_{\mathrm{NL}}$.

To make a direct comparison, we also list the experimental magnetic spin Hall signals measured and corresponding geometrical parameters in these devices, as shown in Supplementary Table 2, where $t_{FGT}$, $t_{MoTe_2}$ and $L$ are the thickness of FGT, the thickness of $MoTe_2$ and the channel length between the FGT/$MoTe_2$ heterostructure region and $MoTe_2$ Hall cross. $\Delta R_{NL}$ and $\alpha$ are the non-local signal and calculated magnetic spin Hall angle, respectively. A significant enhancement of the non-local signal with shorter channel length is observed, which suggests the value of non-local signal is relevant to the channel length $L$. It is noted that the significant magnetic spin Hall signals can be obtained in different devices.

| Sample | $t_{\mathrm{FGT}}$ (nm) | $t_{\mathrm{MoTe_2}}$ (nm) | $L$ (μm) | $\Delta R_{\mathrm{NL}}$ (Ω) | $\alpha$ |
|---|---|---|---|---|---|
| Device 1 | 25.6 | 5.4 | 1.67 | 1.9 | 0.74 |
| Device 2 | 30.8 | 9.4 | 2 | 1.4 | 0.67 |
| Device 3 | 25 | 9.2 | 2.2 | 0.85 | 0.45 |
| Device 4 | 29.1 | 8 | 2.98 | 0.54 | 0.48 |
| Device 5 | 30 | 8.5 | 3.08 | 0.48 | 0.45 |

**Supplementary Table 2.** Summary of devices and devices parameters

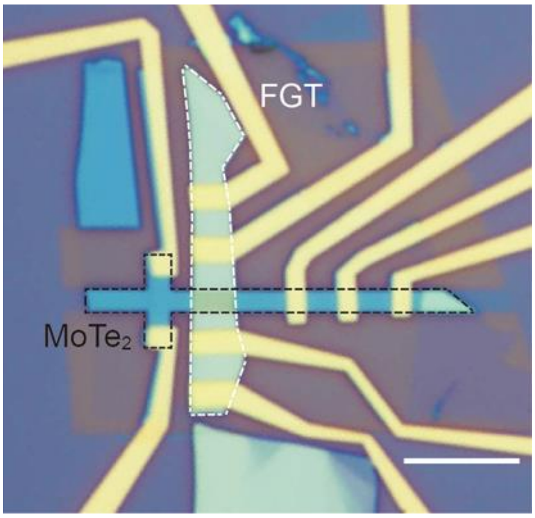


**Supplementary Fig. 3** Optical image of a typical FGT/$MoTe_2$ device. The dashed black lines trace the $MoTe_2$ flake shaped into Hall bar geometry. The dashed white lines trace the FGT flake. Scale bar is 5 $\mu m$.

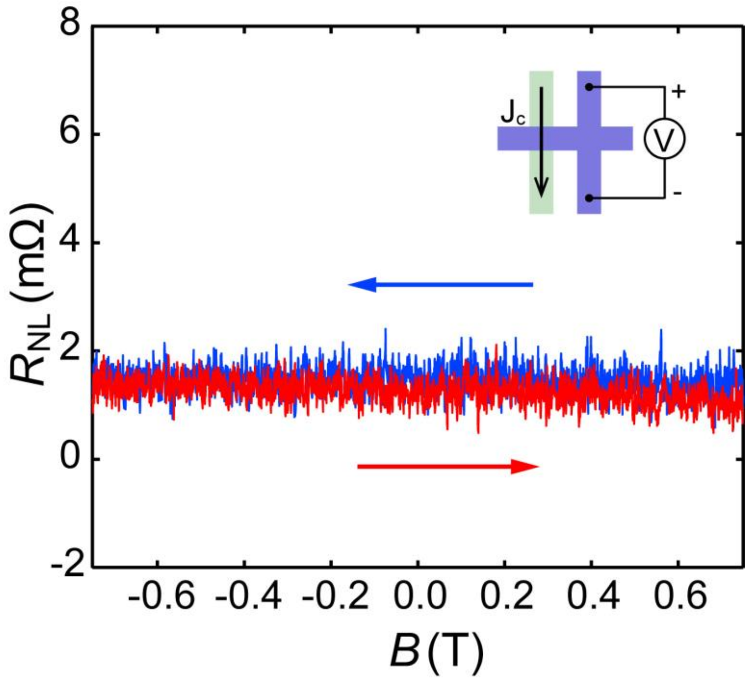


**Supplementary Fig. 4** Non-local measurements in FGT/Graphite device. By using the measurement configuration showed in the inset (same as the measurement configuration adopted in FGT/$MoTe_2$ device), the charge current is applied along FGT and resistance jump measured at graphite Hall cross is absent. This control experiment manifests that any charge diffusion process (such as parasitic anomalous Hall effect) cannot contribute to the $V_{NL}$-$B$ hysteresis loops observed in FGT/$MoTe_2$ device.

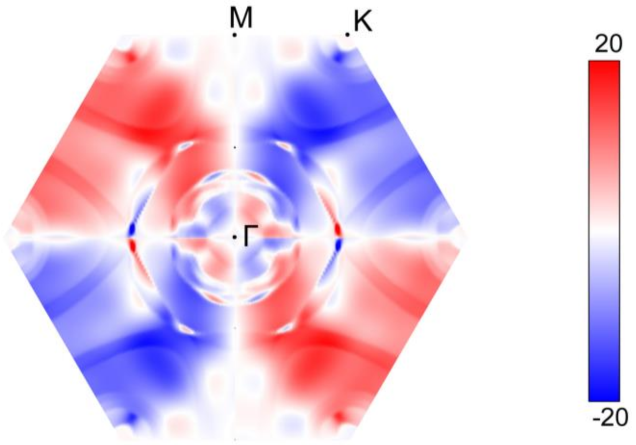


**Supplementary Fig. 5 The calculated *k*-resolved spin current dipole of bulk FGT.** The color code represents the magnitude of the spin current dipole. In bulk FGT, the integration of *k*-resolved spin current dipole $D_{yx}^{S_z}$ in the first Brillouin zone is zero and the corresponding $\mathcal{T}$-odd spin Hall conductivity $\sigma_{yx}^{z}$ is zero, which is consistent with our symmetry analysis.

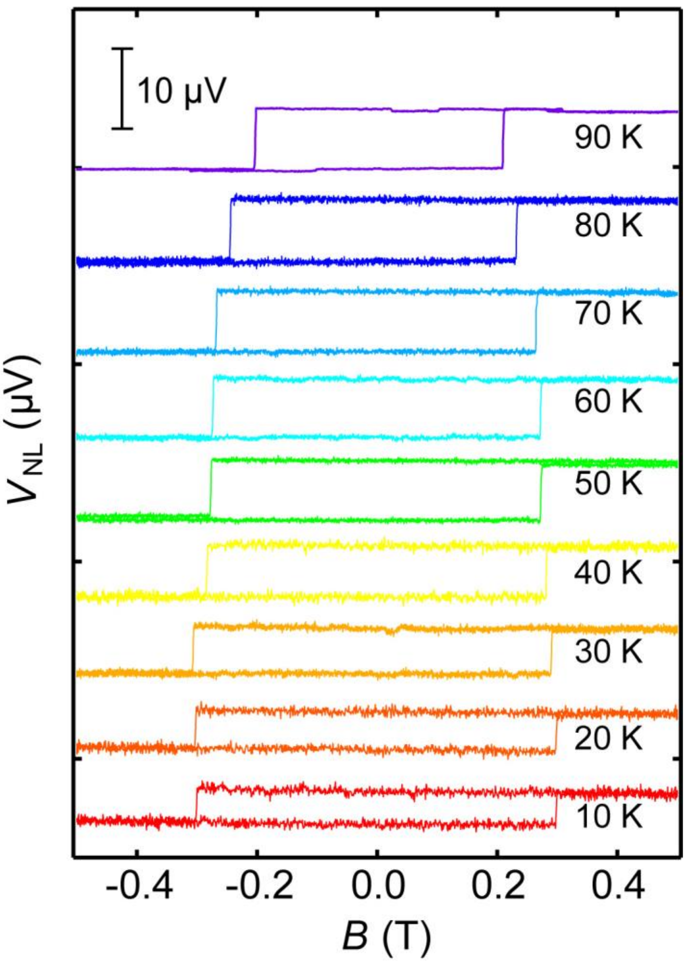


**Supplementary Fig. 6** Measurements of magnetic spin Hall signal at different temperatures. To extract the temperature dependence of spin Hall conductivity originated from the interface of FGT/$MoTe_2$ bilayer, we measure the non-local voltage at different temperatures ranging from 10 K to 90 K. The measurements of non-local voltage as a function of out-of-plane magnetic field are based on the configuration shown in inset of Fig. 2a.

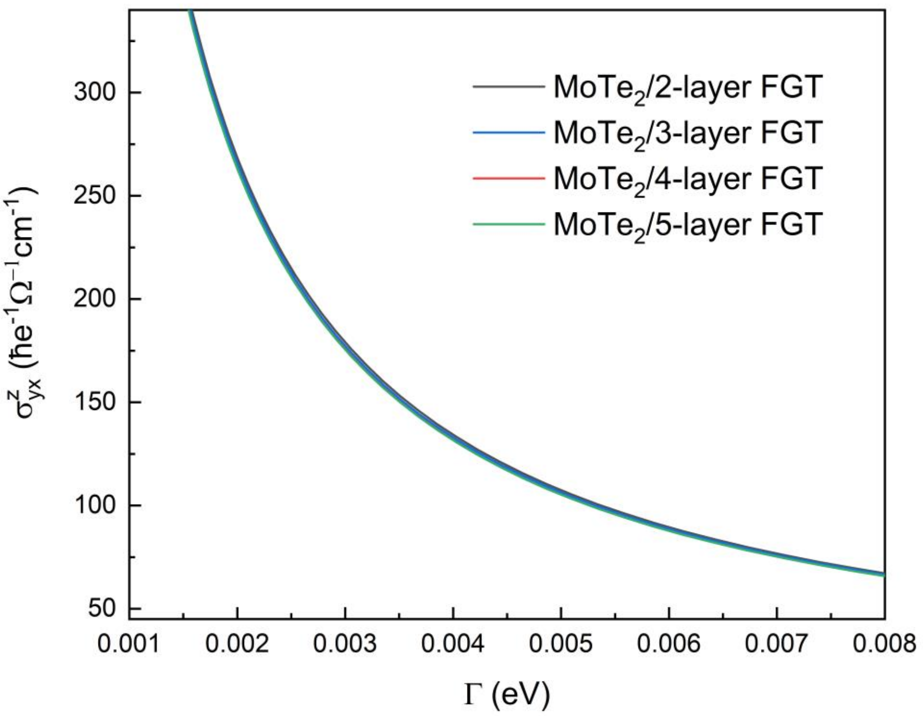


**Supplementary Fig. 7** Calculated $\Gamma$ (defined as $\hbar/2\tau$, with $\tau$ the relaxation time) dependence of $\mathcal{T}$-odd spin Hall conductivity $\sigma_{yx}^{z}$ in heterostructures with different thicknesses of FGT (heterostructures formed by single layer $MoTe_2$ and two/three/four/five layers FGT). The values of spin Hall conductivity in heterostructures with varied thicknesses of FGT are comparable. Meanwhile, the calculated Γ-dependence of the $\mathcal{T}$-odd spin Hall conductivity in these four systems are independent of the thickness, and consistent with our temperature-dependent experimental results, indicating that the magnetic spin Hall effect observed in FGT/$MoTe_2$ heterostructure is an interfacial effect.

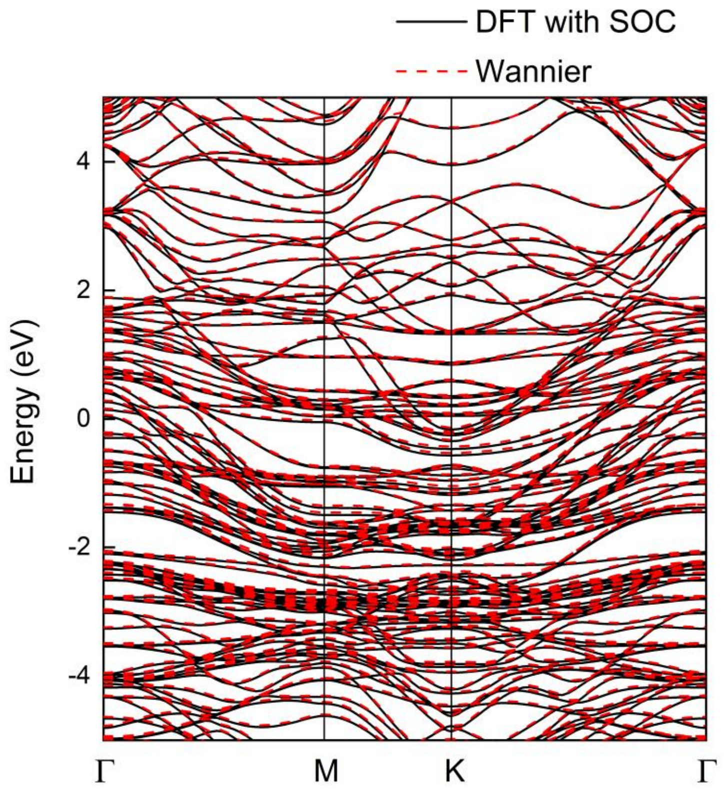


**Supplementary Fig. 8** Calculated electronic structures with spin-orbit coupling (SOC) of bulk FGT in the DFT calculation (black lines) and the interpolation with maximally localized Wannier functions (MLWFs) (red dashed lines). Our calculated result is consistent with previous works (Ref. [6,7]).

## Supplementary References:


1 Abanin, D. A., Shytov, A. V., Levitov, L. S. & Halperin, B. I. Nonlocal charge transport mediated by spin diffusion in the spin Hall effect regime. *Phys. Rev. B* **79**, 035304 (2009).

2 Zhou, J., Qiao, J., Bournel, A. & Zhao, W. Intrinsic spin Hall conductivity of the semimetals $MoTe_2$ and $WTe_2$. *Phys. Rev. B* **99** (2019).

3 Roy, A., Guimarães, M. H. D. & Sławińska, J. Unconventional spin Hall effects in nonmagnetic solids. *Physical Review Materials* **6**, 045004 (2022).

4 Seemann, M., Ködderitzsch, D., Wimmer, S. & Ebert, H. Symmetry-imposed shape of linear response tensors. *Phys. Rev. B* **92**, 155138 (2015).

5 Wimmer, S., Seemann, M., Chadova, K., Ködderitzsch, D. & Ebert, H. Spin-orbit-induced longitudinal spin-polarized currents in nonmagnetic solids. *Phys. Rev. B* **92**, 041101 (2015).

6 Kim, K. et al. Large anomalous Hall current induced by topological nodal lines in a ferromagnetic van der Waals semimetal. *Nat. Mater.* **17**, 794-799 (2018).

7 Jiang, M.-C. & Guo, G.-Y. Large magneto-optical effect and magnetic anisotropy energy in two-dimensional metallic ferromagnet $Fe_3GeTe_2$. *Phys. Rev. B* **105**, 014437 (2022).
